# A preclinical setup for FLASH radiotherapy beam delivery at isocenter using downstream electron scattering

**DOI:** 10.1002/acm2.70582

**Published:** 2026-04-16

**Authors:** Ikechi Ozoemelam, Rowan Paplanus, Michael Pillainayagam, Donald A. Roberts, Dale W. Litzenberg

**Affiliations:** ^1^ Department of Radiation Oncology University of Michigan Ann Arbor Michigan USA; ^2^ Department od Radiation Medicine Oregon Health & Science University Portland Oregon USA

**Keywords:** downstream beam scattering, electrons, FLASH

## Abstract

**Background:**

Preclinical investigations have shown that ultra‐high dose rate (UHDR) radiotherapy (>40 Gy/s) also known as FLASH‐RT, reduces normal tissue toxicity while preserving tumor control efficacy compared to conventional dose rate treatments. Achieving a UHDR‐capable LINAC requires modifications including modifications to electron gun current and bending magnet configurations, use of dedicated energy control boards and use of lower energy scatter foils. However, in the absence of engineering support to implement these changes, it is challenging to realize useful field sizes at ultra‐high dose rates using the upstream scatter foils alone.

**Purpose:**

In this study, we present a configuration to achieve UHDR utilizing downstream electron scattering foils to enable FLASH‐RT at isocenter. By changing the scattering foil position and thickness, we aim to characterize achievable dose rates and dose uniformity for preclinical studies.

**Methods:**

A decommissioned Varian CLINAC 21EX linear accelerator was modified for UHDR irradiation by using the 16 MV beam settings while removing the x‐ray target and flattening filter. Downstream beam scattering was evaluated by testing four lead foil thicknesses (0.28–1.2 mm) at three different source‐to‐foil distances (75.1–90.0 cm), with beam profiles measured using Gafchromic EBT‐XD films at isocenter and evaluated using flatness metrics. The PDDs were also measured to characterize the impact of foils on Bremsstrahlung contribution.

**Results:**

Without a scattering foil, peak dose rates exceeded 200 Gy/s but with poor uniformity (flatness = 22.0%). Standard dual‐foil systems achieved good uniformity (flatness = 6.1%) but yielded non‐UHDR (10–11 Gy/s). With downstream scattering, UHDR (43–135 Gy/s) with improved beam uniformity was achieved. At 75.1 cm source to foil distance, Dmax flatness values at isocenter ranged from 3.5% to 1.2% with increasing foil thickness. However, thicker foils resulted in reduced practical range and increased Bremsstrahlung contribution.

**Conclusion:**

This study demonstrates that downstream electron scattering provides a practical approach for achieving UHDR (43–135 Gy/s) while maintaining acceptable beam uniformity close to the isocenter.

## 1 INTRODUCTION

Preclinical studies have demonstrated that UHDR irradiation (>40 Gy/s), termed FLASH radiotherapy (FLASH‐RT), can significantly reduce normal tissue toxicity while maintaining tumor control, compared to conventional dose rate irradiation.^1^, ^2^, ^3^ Achieving UHDR using electrons requires modifications to standard LINAC configurations. Reported UHDR LINAC modifications have focused on altering LINAC settings, such as increasing the electron gun current, adjusting the bending magnet settings, or using dedicated energy control boards to achieve the desired dose rates.^4^, ^5^ However, the lower dose rates at isocenter often necessitates moving preclinical experimental studies more upstream, posing some inconvenience for reproducible setup.

Conventional electron scattering foil systems were not designed for UHDR beam delivery. The scattering system in an electron LINAC is typically composed of a dual‐foil arrangement with the thickness and material choices predicated on the maximum achievable field size of the LINAC.^6^, ^7^, ^8^, ^9^, ^10^, ^11^ The introduction of scattering material, however, reduces the beam fluence and achievable dose rates. For a 25 × 25 cm^2^ field, the fluence could be reduced by as much as 93% compared to the unmodified beam.^10^ Thus, to obtain dose rates sufficient to elicit the FLASH effect in a field size of about 10 × 10 cm^2^, one might consider using a scattering system designed for a lower beam energy while tuning the beam bending magnet setting to enhance electron transport and increasing the beam current as realized in commercial solutions.^12^, ^13^

In this study, we evaluated a UHDR linac setup that uses downstream electron scattering to spread the beam without modifying beam current or tuning parameters. This approach is particularly valuable for users lacking engineering support. While our approach utilizes typical UHDR configurations including target retraction and flattening filter removal, we replace the engineering‐intensive beam current modifications with downstream scattering foil placement. Downstream scattering foils have the potential to reduce beam divergence and increase the dose rate, although the region having uniform dose might be much smaller than the field sizes for which upstream scattering foils are designed. Our approach addresses a common barrier faced by clinical medical physicists interested in exploring preclinical FLASH studies. The availability of proprietary vendor UHDR packages means that user‐initiated modifications are no longer supported and beam current adjustments and beam tuning under non‐standard configurations require dedicated engineering support. Thus, by empirically changing the position and thickness of the downstream scattering foils, we aim to evaluate the achievable dose rates and uniformity at the isocenter for preclinical studies.

## 2 METHOD

### 2.1 Irradiation facility and LINAC modifications

A recently decommissioned Varian CLINAC 21EX linear accelerator (Varian Medical Systems, Palo Alto, CA, USA) was modified to enable ultra‐high dose rate electron beam delivery. The approach aimed at minimizing the extent of changes made to the LINAC itself, while still achieving the desired UHDR. Previous UHDR LINAC modification approaches have altered various LINAC parameters, such as increasing the electron gun current and adjusting the bending magnet settings^4^, ^5^ or using dedicated energy control boards.^5^ In our setup, the LINAC was configured to use the regular 16 MV beam settings, but with the x‐ray targets and other components removed from the beam path.^14^, ^15^ This was accomplished by reversibly modifying the wiring of the pneumatic system to reverse the logic that drives the target positioning for x‐ray and electron beams. Additionally, the flattening filter was removed by using the manual carousel controller to rotate an empty port into the beam path and, in some cases, replaced with a scattering foil typically used for lower energy electron beams. The collimator jaws and multileaf collimators (MLCs) were fully retracted to allow the maximum field size, ensuring that the only components in the beam path were the vacuum window, the ionization monitor chamber, the mylar mirror, and the beam exit window. The LINAC's servo systems, which normally monitors the beam steering, dose output, and Pulse‐Forming Network (PFN) were disabled, as the ionization chambers used for monitoring become saturated under ultra‐high dose rate conditions.

**FIGURE 1:**
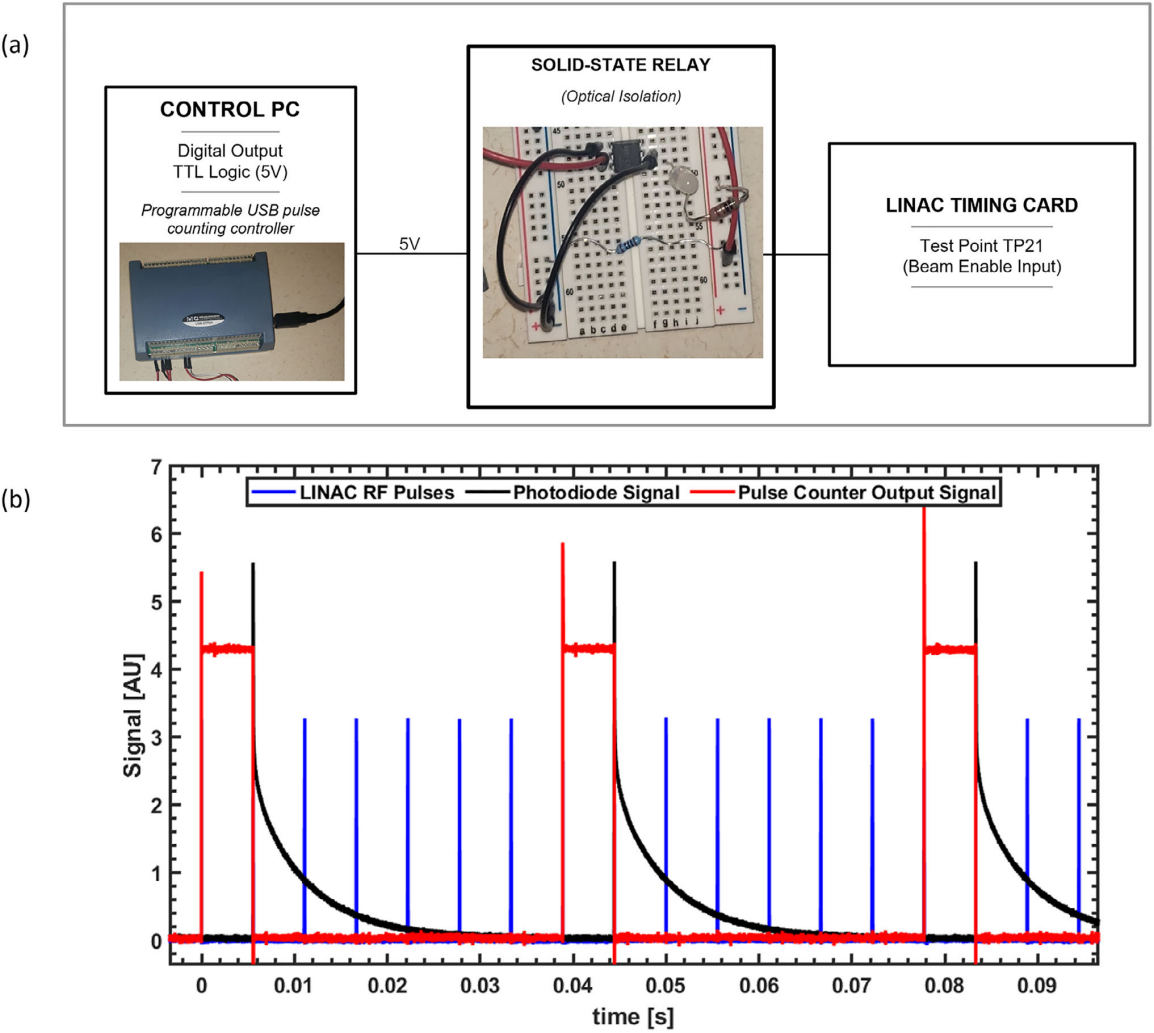
Beam pulsing control setup. (a) Schematic of the external beam control connecting the control PC to the Clinac 21EX timing card via a solid‐state relay. (b) An example of a pulsing control designed to trigger (Pulse counter output) the beam (corresponding to detected photodiode signal) on every 7^th^ Linac RF pulse. Prolonged fall of detected signal is caused by afterglow and not the beam.

To ensure consistent dose delivery on a pulse‐to‐pulse basis, an external control circuit was built and interfaced with the LINAC through the timer interface card. A description of this circuit is provided in Garty et al.^16^ and summarized as follows. The circuit monitors the radiofrequency (RF) waveform of the LINAC to count RF pulses and delivered beam pulses. We modified the timer interface card of the LINAC by providing an external control of the CLINAC's GDLY CNT circuit. Beam delivery was controlled using a USB‐CTR04 counter/timer card (Measurement Computing, Norton, MA, USA). The card receives the KLY I signal from the CLINAC as input. On detecting a user‐specified number of KLY I pulses, the card generates a TTL signal that remains active for a programmable pulse count. This TTL output triggers a solid‐state relay, which modulates the gun delay circuit on the timer interface card to enable or disable beam delivery. Figure 1 shows a schematic of the external beam control setup and a verification of the pulse control using a high‐speed silicon photodetector (DET210, Thorlabs Inc., Newton, NJ, USA) positioned adjacent to an alcohol‐based gel phantom placed in the beam path. The verification shown in Figure 1b represents a scenario where the controller is programmed to trigger the beam on every 7th pulse (25.71 Hz). The coincidence between the rise of the detected photodiode signal and the falling edge of the TTL signal verifies the pulse control. The prolonged signal decay is attributed to delayed emissions from the phantom rather than extended beam duration.

### 2.2 Downstream electron beam scattering

**FIGURE 2:**
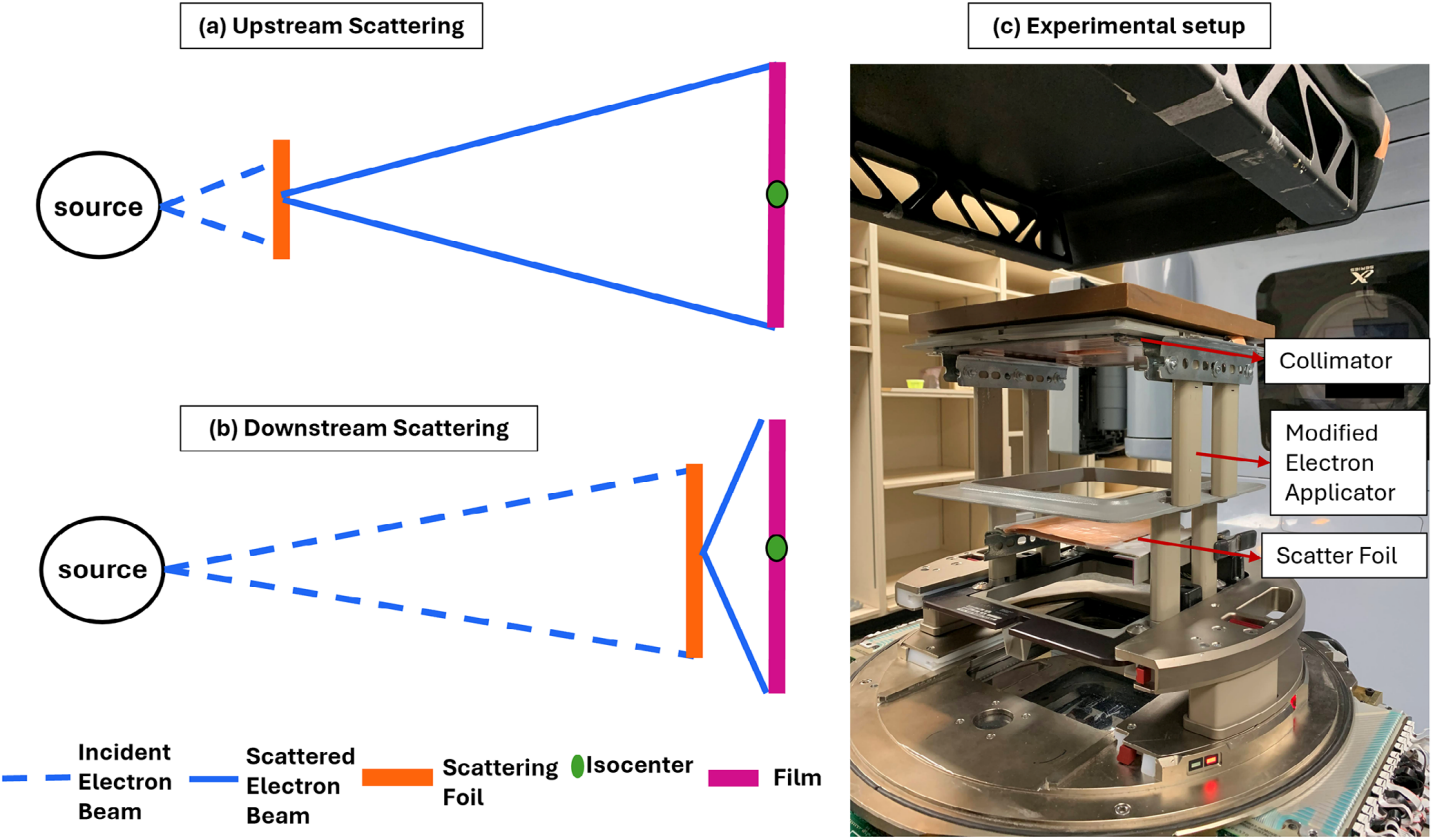
Experimental setup to evaluate downstream scattering. A sketch to show lesser beam divergence resulting from a downstream scattering system (b) as opposed to an upstream one (a). The setup (c) realized with a decommissioned Varian CLINAC 21EX with gantry at 180° and scatter foils positioned using a modified electron applicator.

The use of downstream lead foils for beam scattering, as opposed to the traditional upstream approach, was investigated to reduce beam loss, and optimize the dose rate for UHDR irradiation. Figure 2 shows the setup used in this study. As seen in Figure 2 (Left panel), by placing the scattering foils closer to the target volume, the beam has less distance to diverge, resulting in higher dose rates and improved beam efficiency. To evaluate the impact of downstream beam scattering on the lateral profiles and percentage depth dose (PDD) curves, various configurations of lead foils were investigated and compared to the standard upstream scattering foil system. Lead was chosen as the scattering material due to its practical availability and high scattering power, despite not being the optimal material used in standard LINAC scattering foils. Irradiation was performed with lead foils of four thicknesses: 0.28, 0.4, 0.8, and 1.2 mm. Each foil was placed at three different distances from the source: 75.1, 83.2, and 90.0 cm. The foils were held in position using the electron applicator with a copper collimator at its distal end (Figure 2 right panel). The beam characteristics, including lateral profiles and PDDs, were measured using Gafchromic EBT‐XD film (Ashland Advanced Materials, Bridgewater, NJ) placed between 1 cm thick solid water blocks at the LINAC isocenter (100 cm from the nominal X‐ray source position) for each lead foil configuration. Film dose uncertainty was estimated by combining measurement reproducibility (1%), reference dosimeter calibration (1.5%), post‐irradiation timing variations (1%), and calibration curve fitting (1.5%) in quadrature, yielding a combined standard uncertainty of approximately 2.5%. The dose rates measured in each configuration were obtained as the quotient of the film dose and the duration of the entire pulse train. Ten pulses were delivered for all configurations with a pulse rep rate of 180 pulses/s. The impact on the lateral profiles was evaluated using the flatness and symmetry metric. Flatness is defined as

(1) $$Flatness=\frac{D_{max}-D_{min}}{D_{max}+D_{min}}\;\times 100\%$$

where $D_{max}$ and $D_{min}$ are the maximum and minimum dose, respectively, in the central 80% of a profile drawn in the transverse axis of the field. Beam symmetry was evaluated within the central 80% of the field using the maximum normalized point‐dose difference about the field center of the field:

(2) $$Symmetry=max\left(\frac{\left|D\left(x\right)\;-\;D\left(-x\right)\right|}{D\left(x\right)+D\left(-x\right)}\right)\;\times \;100\%$$

where *D(x)* and *D(−x)* are the dose values at equal and opposite distances from the geometric midpoint of the field.

For comparison, the beam characteristics were also measured using the standard upstream electron scattering foil system. This system consists of a dual‐foil design, with a high‐Z primary foil for initial beam spreading and a low‐Z secondary foil for flattening the beam profile. To assess the impact of the individual foils, measurements were taken with both foils in place and with the lower‐Z secondary foil removed.

## 3 RESULTS

### 3.1 Impact of foil position and thickness on beam profile

**FIGURE 3:**
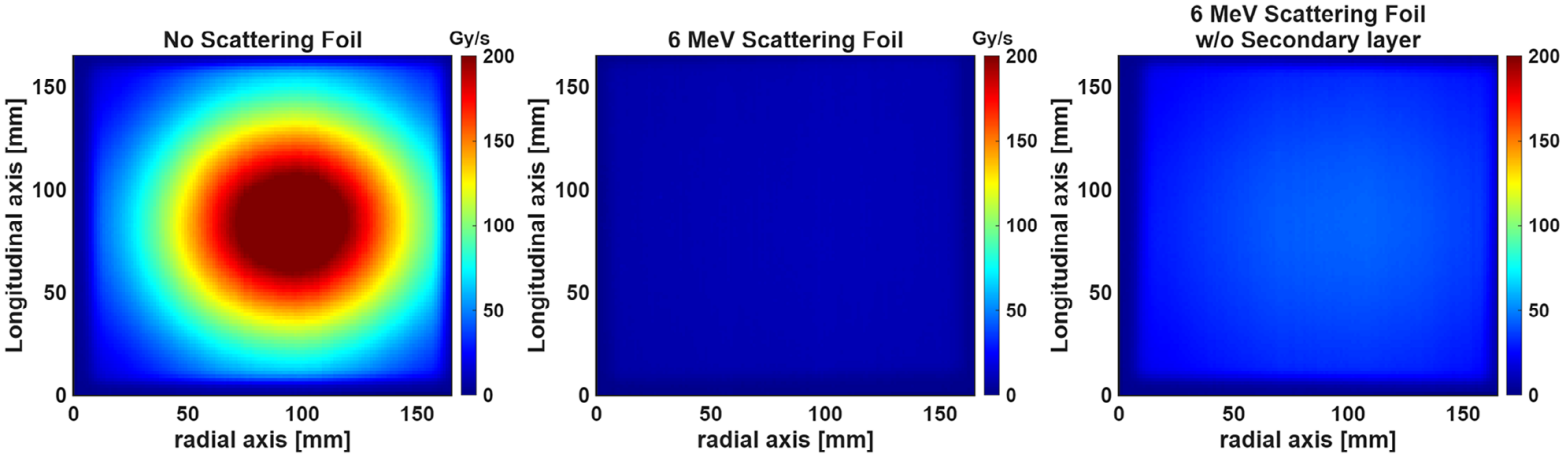
16 MeV electron beam 2D dose rate distribution in 3 scattering configurations. Without any scattering foil (left), using the dual scattering foil of a 6 MeV beam (middle) and the same foil without the secondary foil layer (right). All images are drawn to the same color scale.

**FIGURE 4:**
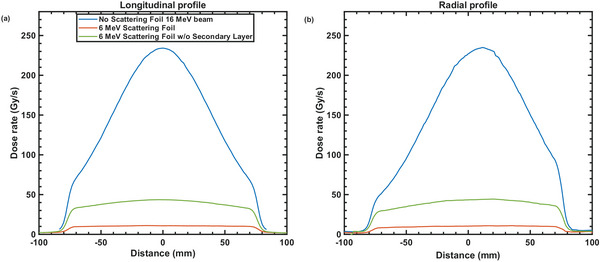
Transverse (a) and radial (b) dose rate profiles extracted from the 2D dose rate distributions shown in Figure 2 for a 16 MeV electron beam under three scattering configurations—no scattering foil, the dual scattering foil designed for a 6 MeV beam, and the 6 MeV primary scattering foil alone without the secondary flattening layer. Profiles are taken along the central axis of each field.

Figure 3 shows the dose rate distribution measured at isocenter for three configurations using a 15 × 15 cm^2^ opening in the copper collimator—open beam, nominal 6 MeV scattering foil and configuration with the secondary layer of the 6 MeV scattering foil removed. The beam without a scattering foil (Figure 3, left panel) showed the highest peak dose rate, exceeding 200 Gy/s in the central region. However, the dose distribution was highly peaked (flatness = 22.0%, transverse/radial symmetry = 1.6%/26.3%), with a pronounced central hotspot and rapid falloff towards the edges of the field. With the full 6 MeV scattering foil in place (Figure 3, center panel), both flatness and symmetry improved (flatness = 6.1%, transverse/radial symmetry = 1.7%/6.5%). However, the dose rate is also significantly reduced, with maximum values around 10–11 Gy/s. This configuration does not meet the minimum 40 Gy/s threshold required for UHDR radiotherapy. Removing the secondary layer of the 6 MeV scattering foil (Figure 3, right panel) resulted in an intermediate scenario. The dose rate increased compared to the full scattering foil, reaching approximately 40 Gy/s in the central region. The dose distribution showed improved uniformity compared to the no‐foil configuration, but has a worse flatness, compared to the full scattering foil, of 11.0% (radial symmetry = 8.6%). The corresponding dose profile in both radial and transverse axis for the three configurations are shown in Figure 4.

**FIGURE 5:**
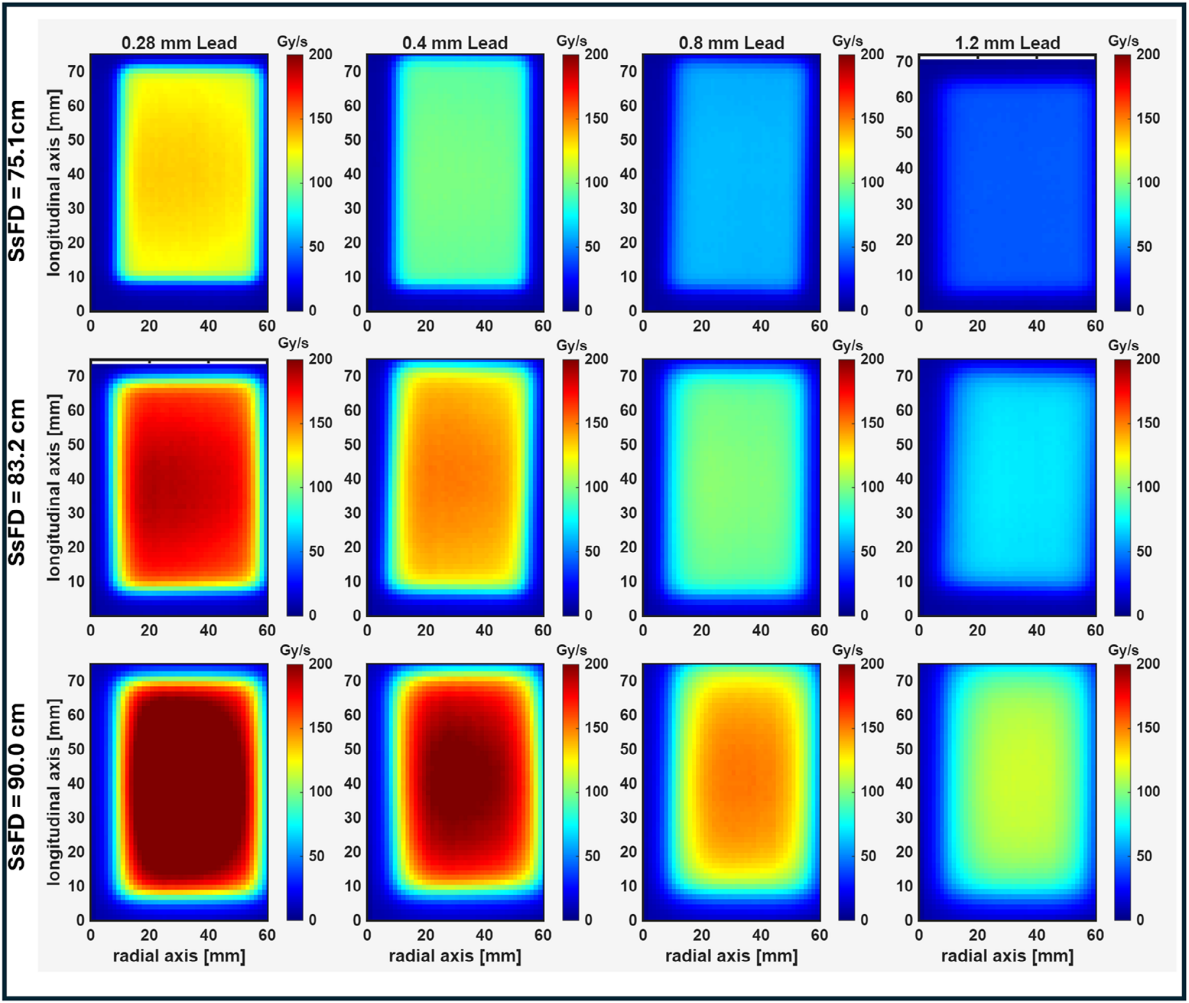
2D dose rate distribution for varying thickness of lead (increasing thickness from left to right) and foils placed at three SsFDs – 75.1 cm (top row) and 83.2 cm (middle row) and 90.0 cm (bottom row). A copper collimator, with a 4 cm × 7 cm opening was placed at the end of the electron applicator. All distributions are shown with the same color scale. The flatness values for each configuration are summarized in Table 1.

**FIGURE 6:**
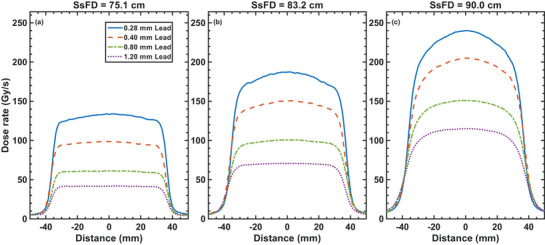
Transverse dose rate profiles for a 16 MeV electron beam scattered through lead foils of varying thickness — 0.28 mm (solid blue), 0.40 mm (dashed red), 0.80 mm (dash‐dotted green), and 1.20 mm (dotted purple) — at three source‐to‐scattering‐foil distances (SsFD): 75.1 cm (a), 83.2 cm (b), and 90.0 cm (c). Profiles are extracted from the 2D dose rate distributions shown in Figure 4 along the central transverse axis.

To achieve both UHDR and improved dose uniformity, we investigated the use of downstream scattering foils placed at different positions from the isocenter. Figure 5 shows the dose rate distributions for lead foils of varying thicknesses (0.28, 0.4, 0.8, and 1.2 mm) positioned at three different source‐to‐scattering‐foil distances (SsFDs): 75.1, 83.2, and 90.0 cm. The corresponding dose rate profiles along the transverse axis is shown in Figure 6. Due to the asymmetry of the beam, a copper collimator, with a 4 cm × 7 cm opening off‐centered from the central axis in the radial direction, was used for each configuration. Table 1 summarizes the flatness and maximum dose rates within the field for each configuration. The transverse symmetry for all configurations is within 1.5%. The results show that at SsFD = 75.1 cm (closest to the source), the flatness values are generally lower, indicating better uniformity. Increasing the thickness of the foil results in better flatness. However, as the SsFD increases, the flatness worsens. This trend is consistent across all foil thicknesses. These results demonstrate that the choice of foil thickness and position impacts the beam flatness. The best uniformity is achieved with thicker foils at the closest SsFD (75.1 cm). However, this needs to be balanced against the achievable dose rate. The gain in flatness by a factor of 2.9, when changing the foil thickness from 0.28 to 1.2 mm at an SsFD of 75.1 cm, results in a drop of maximum dose rate by the same amount. The dose rate distributions in Figure 5 visually confirm these trends, showing more uniform fields for thicker foils and at closer SsFDs. The corresponding dose profile in the transverse axis for all 12 configurations are shown in Figure 6.

**TABLE 1:** Flatness and maximum dose rates within field for various configurations. Only flatness in the transverse axis is shown.

	0.28 mm Pb		0.4 mm Pb		0.8 mm Pb		1.2 mm Pb	
Foil Position [cm]	Flatness [%]	Max Dose rate [Gy/s]	Flatness [%]	Max Dose rate [Gy/s]	Flatness [%]	Max Dose rate [Gy/s]	Flatness [%]	Max Dose rate [Gy/s]
75.1	3.5	134.9 ± 3.4	2.4	99.4 ± 2.5	2.1	62.2 ± 1.6	1.2	42.8 ± 1.1
83.2	5.9	191.0 ± 4.8	4.8	151.6 ± 3.8	3.6	102.3 ± 2.6	3.7	71.7 ± 1.8
90.0	8.5	243.4 ± 6.1	8.6	207.8 ± 5.2	8.4	152.4 ± 3.8	8.2	115.7 ± 2.9

### 3.2 Depth dose

**FIGURE 7:**
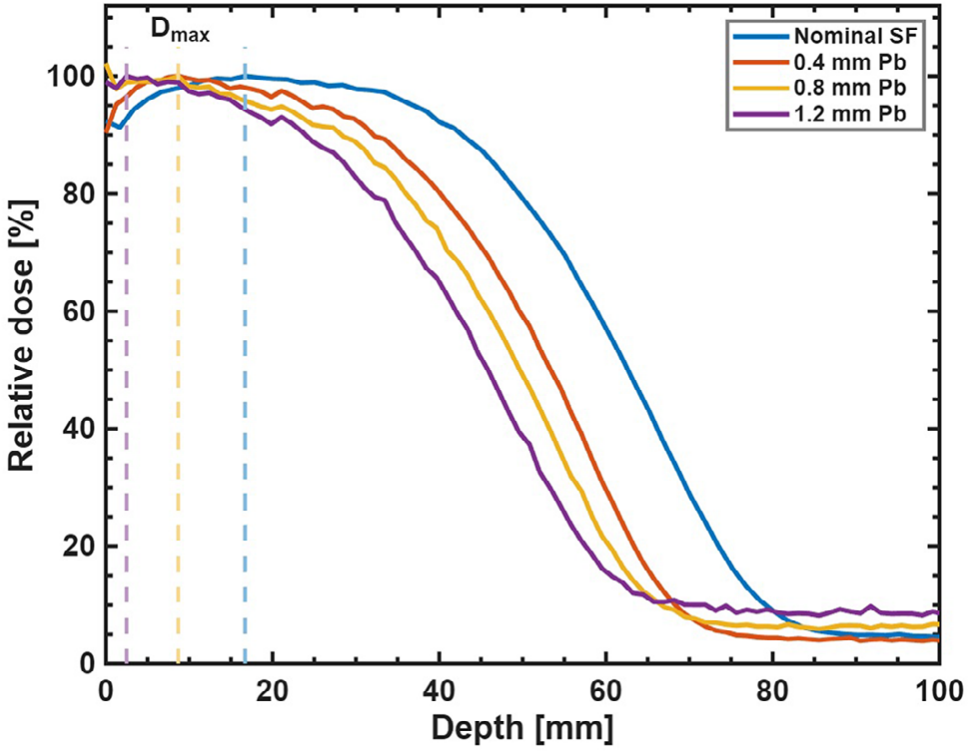
Depth dose profiles obtained with various thickness of lead foils, at 77.1 cm SsFD, compared to nominal upstream scattering foil. The dash lines indicate the depth of Dmax for each configuration.

To evaluate the effect of downstream scattering foils on the electron beam's depth‐dose characteristics, we measured Percentage Depth Dose (PDD) curves for various lead foil thicknesses. Figure 7 shows the PDD curves for the nominal scattering foil (SF) and lead (Pb) foils of 0.4, 0.8, and 1.2 mm thickness. As the lead foil thickness increases, there is a notable shift in the practical range of the electron beam towards shallower depths. The nominal SF configuration shows the deepest penetration, while the 1.2 mm Pb foil results in the shallowest penetration. This shift is due to the increased energy loss and scattering of electrons in thicker lead foils. A further consequence of increasing lead thickness is an increase in the Bremsstrahlung contributions. The nominal SF configuration has the lowest Bremsstrahlung component. As lead thickness increases, there is a slight increase in the Bremsstrahlung tail intensity, with the 1.2 mm Pb foil showing the highest Bremsstrahlung contribution by a factor of 1.9 compared to the nominal scattering foil.

## 4 DISCUSSION

In this study, a preclinical UHDR radiotherapy setup that integrates downstream electron scattering for uniform isocentric dose distribution was evaluated. The results demonstrate that UHDR can be achieved while maintaining a uniform dose radiation field using the downstream scattering setup. Notably, thicker lead foils positioned closer to the source (75.1 cm SsFD) provided the best balance between uniformity and dose rate. This finding offers a potential solution to two common scenarios encountered when attempting to convert a clinical LINAC to a UHDR‐capable machine for experimental studies. The first scenario relates to the limitations faced by users who are unable to modify the electron gun current or lack the engineering support necessary to tune the steering magnets with further increases in beam current. Some approaches to achieving UHDR often rely on substantial modifications to the LINAC's electron gun or beam steering systems,^4^, ^13^ which may not be feasible in all clinics. The second scenario pertains to experimental setups where users are unable to move their apparatus closer to the electron source to exploit the inverse square effect for achieving higher dose rates. Many UHDR radiotherapy studies have relied on positioning samples very close to the vacuum window to achieve the required dose rates,^5^, ^17^ but this approach often comes with limitations in terms of field size, uniformity, and practical experimental setup. The current study's approach of using downstream scattering foils at a more practical distance (75.1 cm SsFD) demonstrates that UHDR can be achieved while maintaining a more conventional experimental geometry, precluding the otherwise necessary modifications. The simplicity of our approach could lower the barrier to entry for UHDR radiotherapy research, allowing a broader range of institutions to participate in clinical translation of FLASH‐RT. Furthermore, the ability to achieve both UHDR and improved uniformity at isocenter distances more typical of clinical setups (closer to 100 cm SSD) represents a significant step towards translating FLASH‐RT from purely experimental settings to potential clinical applications.

Although the current study used lead foils, which may not be optimal in terms of achievable thickness, it provides proof of concept for the downstream scattering approach. Future research would explore more suitable materials, thickness and configurations to further optimize this technique. However, it is important to note that this approach does come with its own set of challenges. The trade‐off between improved uniformity and reduced field size compared to conventional upstream scattering foils needs to be carefully considered. Additionally, the impact of the downstream scattering foils on the depth‐dose characteristics and Bremsstrahlung production will require careful consideration in treatment planning and dosimetry, though this problem might be minimal if thinner thickness of more optimal materials and a double layer configuration is used.^8^, ^10^

## 5 CONCLUSION

In conclusion, we have demonstrated, in this proof‐of‐concept study, that the use of a novel UHDR radiotherapy setup that integrates downstream electron scattering for uniform isocentric dose distribution achieves UHDR while maintaining acceptable beam uniformity at relevant distances from the source. The downstream scattering approach offers a promising solution for achieving UHDR with minimal modifications to existing linear accelerators. This study lays the groundwork for future investigations into more optimal materials and configurations for beam shaping.

## AUTHOR CONTRIBUTIONS

Ikechi Ozoemelam was involved in study design, data collection, data analysis, and drafting of the manuscript. Both Rowan Paplanus and Michael Pillainayagam were involved in data collection, data analysis and manuscript review. Donald Roberts was involved in study design and manuscript review. Dale Litzenberg was involved in study design, data collection, organization and structure of the manuscript, overall review of the manuscript and supported the interpretation of representational data. All authors read and approved the final version of the manuscript.

## FUNDING INFORMATION

No funding sources to disclose.

## CONFLICT OF INTEREST STATEMENT

The authors declare no conflicts of interest.

## Data Availability

Authors will share data upon reasonable request to the corresponding author.
